# Comparing the Effect of Piperine and Ilepcimide on the Pharmacokinetics of Curcumin in SD Rats

**DOI:** 10.3389/fphar.2021.725362

**Published:** 2021-10-29

**Authors:** Peijian Wang, Hui Li, Zhexuan Lin, Hongjun Luo, Wenhong Luo

**Affiliations:** Bio-analytical Laboratory, Shantou University Medical College, Shantou, China

**Keywords:** curcumin, piperine, ilepcimide, plasma concentration, pharmacokinetics, dihydrocurcumin

## Abstract

The poor bioavailability and rapid metabolism of curcumin (CUR) restrict its clinical application. Piperine (PIP), which was extracted from natural compounds, can increase the plasma concentration of curcumin in humanidad. As an artificial synthetic piperine analog, silepcimide (ILE) has significant advantages because of the low price and simple synthesis process. In this study, a simple and rapid HPLC-UV method was developed for determination of the plasma concentration of CUR, PIP,ILE and dihydrocurcumin (DHC, a metabolite of CUR) simultaneously. Meanwhile, the effects of PIP and ILE on the plasma concentration and pharmacokinetics of DHC in SD rats was studied to explore whether ILE could serve as a CUR bioavailability enhancer. The metabolic pathway of CUR was studied by comparing the differences of CUR plasma concentration between intravenous injection and oral administration over the same time period, and reacting with small intestine homogenate without microbes of SD rats. The results of drug-time curve showed that combined administration of ILE and CUR had significant effect on plasma concentrations of DHC. Repeated administration of PIP or ILE could significantly increase the plasma concentration of DHC. Plasma CUR could be detected in the samples of from intravenous injection of CUR rats, whereas, it couldn’t be detected in the plasma sample form oral administration rats. CUR incubated with intestinal homogenate without intestinal bacteria could not be transformed into DHC. In conclusion, our results show that ILE can improve the bioavailability of CUR. Additionally, it was inferred that most of the CUR was reduced to DHC by NADPH when it was absorbed from gastrointestinal tract, and our results demonstrated that this pathway might be mediated by gastrointestinal microorganisms.

## Introduction

Curcumin (CUR) is a natural diketone compounds which is extracted from curcuma rhizome (Aggarwal et al., 2007). Since two thousand BC, turmeric has been widely used in Asian medicine (Sharma et al., 2005). Besides its aromatic, stimulant and colouring properties in diet (Eigner and Scholz, 1999), curcumin can inhibit the activity of oxidative stress and the activity of nuclear factor kappa-B(NF-κB), Cyclooxygenase-2(COX-2), lipoxygenase(LOX), inducible NOS)iNOS) and other inflammatory mediators, thereby plays an anti-inflammatory and anti-oxidant role (Bengmark, 2006; Li J. et al., 2019). In terms of clinical treatment, curcumin is used for the treatment of atherosclerosis (Li X. et al., 2019), tumor (Asensi et al., 2011; Purkayastha et al., 2009), diabetes (Zhao et al., 2014), inflammatory bowel disease (Holt et al., 2005), neurodegenerative diseases (Abdenour et al., 2011; Darvesh et al., 2012; Ullah et al., 2017) and malaria (Reddy et al., 2005; Nandakumar et al., 2006). Recently, curcumin was reported that it could prevent SARS–CoV–2 entry into the cells and protect and repair the damage of alveolar cells, renal cells, cardiomyocytes, hematopoietic stem cells induced by COVID-19 (Soni et al., 2020). Therefore, clarification of the pharmacokinetics of curcumin and its related factors would improve its clinical application, which is of great important in the research and development of natural products. Meanwhile, establishment of an efficient and convenient method for the determination of curcumin in biological samples would be helpful for the study of curcumin pharmacokinetics.

Curcumin has low polarity specificity and is insoluble in water and various vegetable oils (Chang et al., 2013). The oral bioavailability of curcumin is only 1% (Yang et al., 2007), and most of it is excreted in the prototype form by feces (Wahlström and Blennow, 2010; Holder et al., 1978). Nanometer preparations of curcumin can improve its bioavailability, but the high cost and tech in this process restrict its wide application in animal experiments (Mohanty and Sahoo, 2010).

Dihydrocurcumin (DHC) is an intermediate metabolite of curcumin in the body, which has the physiological effect of reducing lipid accumulation and oxidative stress (Yu et al., 2018). It has been shown that curcumin can be metabolized to dihydrocurcumin and tetrahydrocurcumin (THC) by NADPH-dependent enzymes in gut microbiota (Hassaninasab et al., 2011). Curcumin undergoes rapid Phase I (reduction reactions) and Phase II (conjugation reactions) metabolism mainly in the liver, intestines, and gut microbiota. Dihydrocurcumin, tetrahydrocurcumin and hexahydrocurcumin derive from curcumin by Phase I reductive metabolism, and then transform into corresponding glucuronide-conjugates by phase II metabolism (Asai and Miyazawa, 2000; Pan et al., 1999).

Piperine (PIP) is a natural compound extracted from the mature seeds of black pepper, which can be used as flavorings in food depending on its biting taste (Smilkov et al., 2019). It also has many physiological effects, such as antioxidant (Khajuria et al., 1998), anti-tumor (Pradeep et al., 2002; Selvendiran et al., 2004), anti-allergy (Qiao et al., 2020), anti-depression (Li et al., 2007), anti-epilepsy effect (Ren et al., 2019a; Ren et al., 2019b) and so on. Additionally, piperine is a bioavailability enhancer, which can increase bioavailability when used in combination with rifampicin, phenytoin sodium, verapamil, and et al.(Bhardwaj et al., 2002), by preventing the drug efflux from cells or reducing the metabolism of drugs (Bakshi et al., 2021). When curcumin are administrated together with piperine, the blood concentration of curcumin and metabolic time can be increased leading to the improvement of effect (Shoba et al., 1998). The specific mechanism is that piperine is an inhibitor of glucuronic and curcumin is metabolized through glucuronic in the body (Asai and Miyazawa, 2000; Pan et al., 1999; Dogra et al., 2004).

Pepper has been used for the treatment of epilepsy since the Tang Dynasty in China. In the 1970s, Beijing Medical College developed a new drug, which was later called “Ilepcimide” (ILE). It was an analogue of piperine, whose cinnamon amides was substituted by aromatic ring, and served as antiepilepsirin with low toxicity.

In the 1990s, the production of ILE was halted due to difficulties in supplying raw materials and high manufacturing costs. It was not until 2009 that the drug was remanufactured with the name of “Ilepcimide (ILE)”. There has been little research on Ilepcimide so far. Ilepcimide is a derivative of piperin, with one less hydrocarbon group than piperin in structure. Both of them exert antiepilepsy effect. However, the similarity of other pharmacological effects is still unclear. Further research is needed to determine whether Ilepcimide could increase the blood concentration of curcumin as well as piperine. Ilepcimide is a synthetic drug, which has the advantages of simple synthesis process, and low price. If it could increase the bioavailability curcumin, it would be beneficial to solve the shortcoming of low oral bioavailability and short half-life of CUR.

## Materials

### Chemicals and Reagents

Curcumin (CUR) and Piperine (PIP) were purchased from Xi ‘an Yunyue Biological Technology Co., Ltd. Ilepcimide (ILE) were purchased from Beijing SCRIANEN Co., Ltd. Dihydrocurcumin (DHC) were purchased from Shanghai Yuanye Biotechnology Co., Ltd. Tetrahydrocurcumin (THC) were purchased from Aladdin. Acetonitrile (ACN) and Methanol (MeOH)were purchased from Merck. Vitamin C(Vc)、Acetic acid (HAc) and dimethyl sulfoxide (DMSO) were purchased from Aladdin. CURPIP, ILE, DHC, THC and Vc were of analytical grade. ACN, DMSO, MeOH and HAc were of chromatographically pure grade.

**FIGURE 1:**
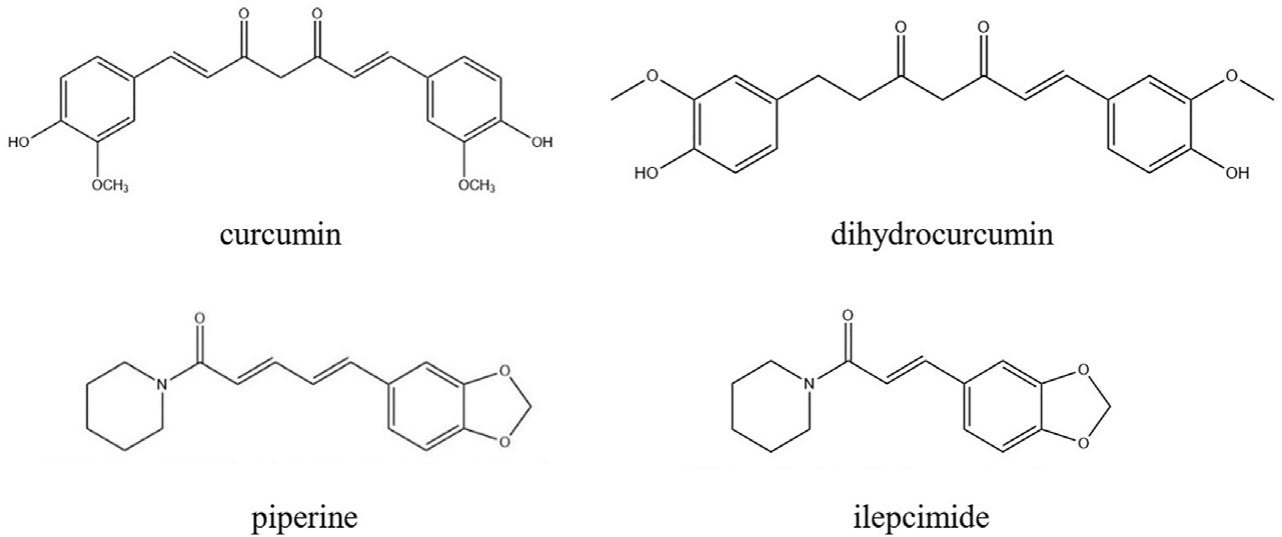
Chemical construction of CUR, DHC, PIP and ILE.

ILE was used as internal standard for the detection of CUR, PIP and DHC. PIP was used as internal standard for the detection of CUR, ILE and DHC (Figure 1).

### Animal

Specific pathogen-free (SPF) grade healthy male Sprague–Dawley (SD) rats (n = 60, 6 week old) weighing 300–360 g were purchased from Hunan SJA Laboratory Animal Co., Ltd. They were housed in Laboratory Animal Center of Shantou University Medical College with water and food intake freely. The experiments were performed after 12 h fasting. The animal experiment procedure was approved by the Animal Experiment Ethical Inspection of Shantou University Medical College No. SUMC 2020-344.

## Methods

### Analytical Method

#### Chromatographic Condition

An Agilent 1200 HPLC system was used. ILE, CUR, PIP and DHC were separated simultaneously by using Gemini-NX New Column (250 mm × 4.6 mm×5 μm) with the mobile phase of ACN:2% HAc in water (58:42, v/v). The flow rate was set at 1 ml min^−1^ and detection wavelength was 420, 340 and 323 nm. Injection volume was 5 μL. The column temperature was set at 30°C.

#### Preprocessing of Samples

In order to optimize the efficiency of protein precipitation, different ratios between CAN and MeOH were studied (n = 3). Then, The protein precipitant with 90% ACN:10% MeOH (1% HAc) was develop to precipitate the plasma protein and release the drugs form plasma proteins, resulting in the highest recovery rate of target drugs.

Plasma or brain homogenate of SD rats was taken 50 μL, internal standard solution of 50 μL and protein precipitator of 150 μL were added. After eddy oscillation for 3 min, supernatant was separated by centrifugation.

#### Calibration Curves

Stock solutions of CUR, PIP and ILE were prepared in ACN. Serial dilutions of the stock were carried out in ACN. Calibration standards were prepared by spiking with known amounts of the working solutions of CUR, PIP, ILE and DHC in plasma. Quality control samples were prepared by spiling known amounts of CUR, PIP, ILE and DHC at three concentration levels into human plasma.

#### Methodology Validation

##### Limits of Detection and Limit of Quantitation

LOD is equivalent to three times the height of the average baseline noise, while LOQ is the concentration at 10 times the peak height of baseline noise (Agency, 2006). After the blank samples of plasma and brain homogenate were detected, the baseline chromatogram was enlarged, and the noise heights of five points were randomly selected to get the average value. Then, according to the standard curves of plasma samples and brain homogenate samples, LOD and LOQ of this method were calculated form three times the noise height and 10 times the noise height respectively.

##### Intra-day Precision and Inter-day Precision

The precision and accuracy of the method were assessed on the same day (intra-day) and on three consecutive days (inter-day). Briefly, the mixed standard solution of CUR, PIP and ILE with 0.05, 0.2 and 1.0 μg mL^−1^ was added into blank plasma and brain homogenate of SD rats (each with three replicates). After processing the samples according to the method of plasma and brain homogenate in 3.1.2, the peak areas of the three compounds were determined and the recoveries were calculated.

$$Recoveries=\frac{\text{Peak Area of sample }\left(\text{plasma or brain homogenate }\text{sample adding standard solution}\right)}{\text{Peak Area of blank control}\left(\text{standard }\text{solution of the same concentration}\right)}.$$

##### Stability Experiment

Three blank samples of plasma and brain homogenate of SD rats were added into the mixed standard solution. The stability experiment included placing at room temperature for 12 h, repeatedly freeze-thaw for three times and frozen for 15 days. Then the concentration of CUR, PIP and ILE were determined as described in 3.1.2, and the recovery were calculated to assess the stability of the sample.

### *In vivo* Experiment

#### Qualitative Analysis of Metabolin of CUR

The retention time of CUR standard was 5.9 min. However, during the experiment, the chromatographic peak of CUR was not observed at wavelength of 420 nm by using ultraviolet detection, but a new chromatographic peak appeared at the retention time of 3.0 min. The chromatographic peak did not exist in blank plasma, and the changes of peak area showed an administration dose-dependent and time-dependent manner. Therefore, we speculate that it is the metabolite of curcumin. The metabolites were qualitatively analyzed by mass spectrometry and compared with the retention time of DHC and THC.

#### The Solvent and Dose of Intragastric Administration

CUR is insoluble in water and various plant extracted oils, and slightly soluble in EtOH and glycerin, which might influence the absorption after intragastric administration without complete dissolvement in the vehicles. DMSO is a universal organic solvent that is mutually soluble with most solvents and has a protective effect on red blood cells, platelets, bone marrow and various tissue cells during cryopreservation (Willson et al., 1965). CUR, PIP and ILE are well dissolved in DMSO, making it easy to control the drug dose by intragastric administration. The LD50 of intragastric administration of DMSO in rats was 28.3 g kg^−1^, which was much higher than the dose we used in this experiment. Therefore, the use of DMSO as an intragastric dose in animal experiments does not cause toxicological and pathological changes in SD rats. The results showed that the mixture of 80% DMSO and 20% EtOH was used as gavage agent with CUR at dose of 200 mg kg^−1^, the concentration of curcumin metabolites in rat plasma could be greatly improved. The oral doses of the three drugs were as follows: CUR 200 mg kg^−1^, PIP 150 mg kg^−1^, and ILE 150 mg kg^−1^.

#### Methods for Pharmacokinetic Experiments

SD rats were randomly divided into three groups: A(CUR), B(CUR and PIP), C(CUR and ILE). The rats were given oral administration with CUR 200 mg kg^−1^, PIP 150 mg kg^−1^, ILE 150 mg kg^−1^ (dissolved in 80% DMSO and 20% EtOH). Blood samples were collected from tail vein at 20 min, 40 min, 1, 1.5, 2.5, 3.5, 4.5, and 5.5 h after intragastric administration.

The concentration of drugs in plasma and brain homogenate after repeated administration was determined according to the same grouping and administration method. The loading dose of 200 mg kg^−1^ was given for the first time and half doses for four consecutive days. Blood was collected from inferior vena cava and brain tissue was also collected.

Plasma was collected by centrifugation of blood at 3000 RPM for 10min, and stored at -80 °C. Brain tissue of SD rat was dissected, weighed and added with normal saline at a volume of 2 ml g^−1^. After homogenating brain tissue for 3 min by a homogenizer at 14,000 RPM, the supernatant was collected after centrifugation at 12,000 RPM for 10 min.

#### Absorption and Metabolism of Curcumin in SD Rats

SD rats were injected with CUR through tail vein, and 20 min later, blood was collected from tail vein to detect drug concentration, comparing the differences of plasma CUR concentration between intravenous injection and oral administration after the same time.

The metabolic pathway of CUR was studied by incubation with small intestine homogenate without microbes of SD rats.

## Results

### Analytical Method

**FIGURE 2:**
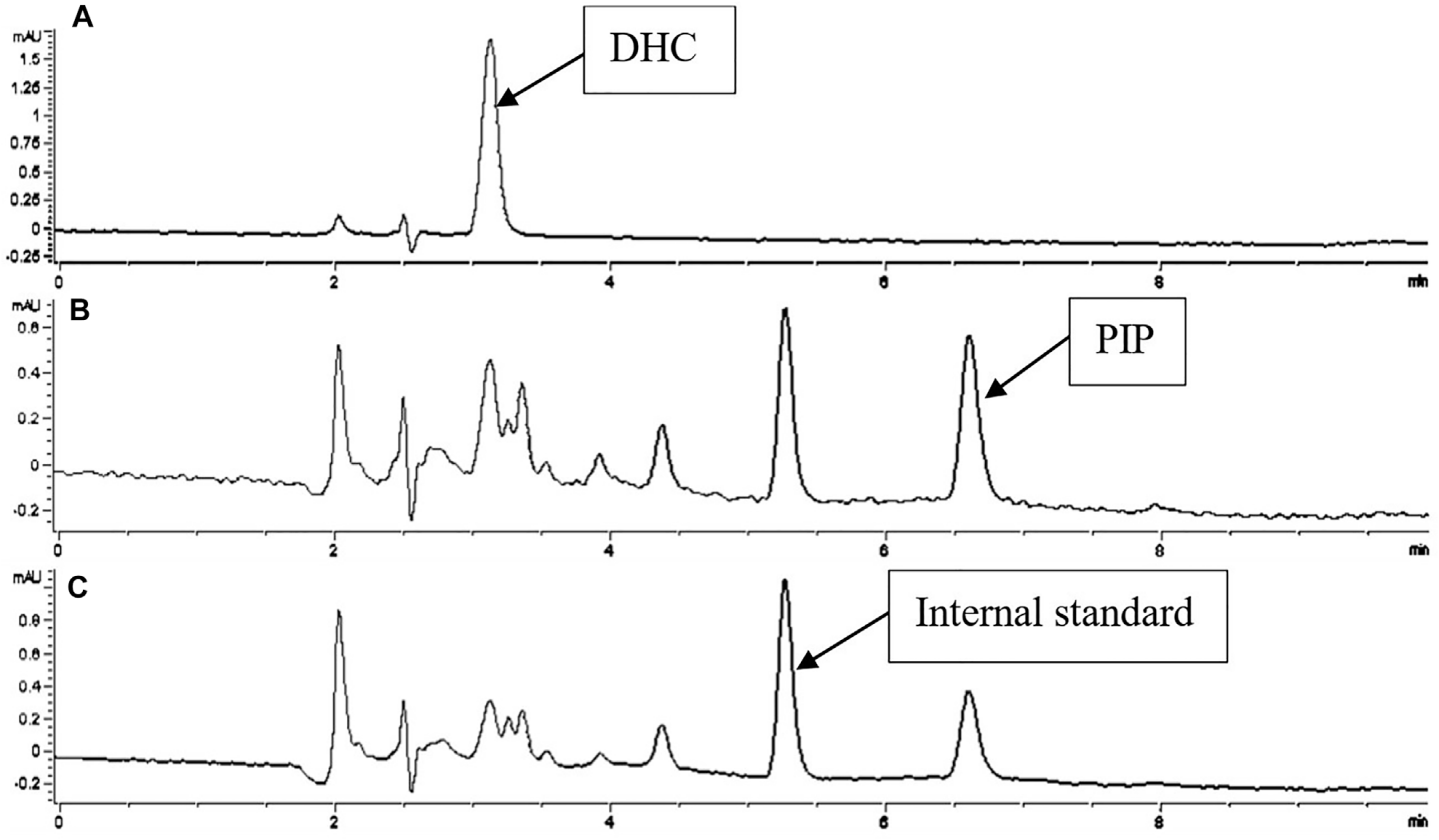
Chromatogram of DHC and PIP in biological samples with ILE as internal standard. Detections of A, B, C were recorded at 420, 340, 323 nm.

**FIGURE 3:**
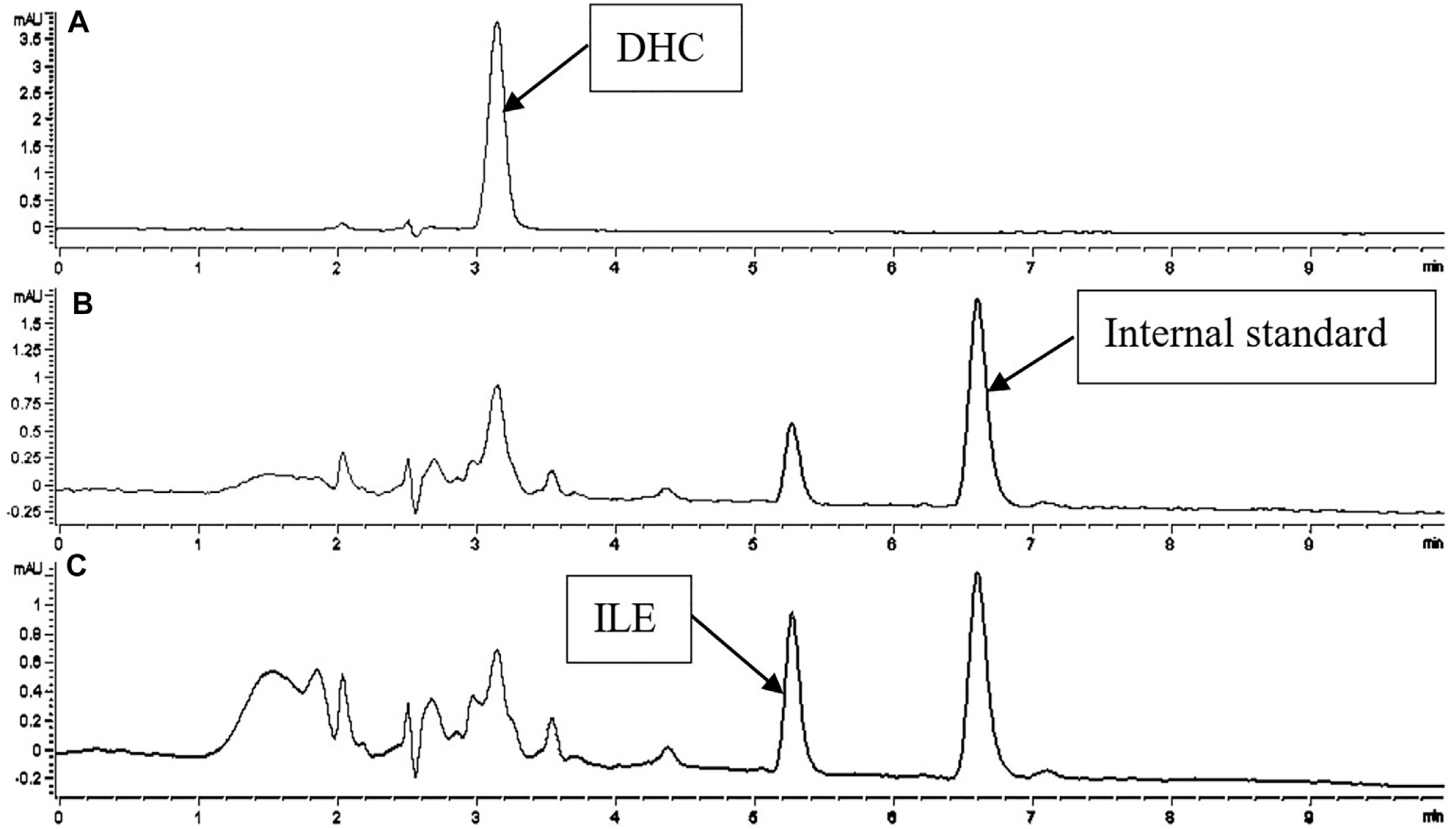
Chromatogram of DHC and ILE in biological samples with PIP as internal standard. Detections of A, B, C were recorded at 420, 340, 323 nm.

A simple and rapid HPLC-UV method was developed, which could simultaneously determine the concentration of CUR, PIP, ILE and DHC in the plasma and brain homogenate (See Figures 2, 3). 90% ACN and 10% MeOH (1% HAc) were used as protein precipitator for pretreatment, which had the advantages of simple operation and high recovery (Table 1).

**TABLE 1:** Efficiency of protein precipitator (n = 3).

**Protein precipitant (v/v)**	**Mean recoveries of CUR ± sd (%)**	**CV (%)**
MeOH	7.5 ± 0.5	6.2
ACN	14.9 ± 1.6	10.6
MeOH (5 mg·mL^−1^ Vc)	69.4 ± 2.6	3.8
50% ACN∶50% MeOH (5 mg·mL^−1^ Vc)	87.9 ± 1.7	1.9
60% ACN∶40% MeOH (5 mg·mL^−1^ Vc)	96.0 ± 1.4	1.5
60% ACN∶40% MeOH	30.3 ± 5.1	16.8
70% ACN∶30% MeOH	70.1 ± 1.2	1.6
80% ACN∶20% MeOH	85.9 ± 1.3	1.5
90% ACN∶10% MeOH	89.6 ± 0.9	1.0
90% ACN∶10% MeOH (1% HAc)	102.6 ± 1.7	1.7

Vc, Vitamin C.

In this experiment, the internal standard method is used to draw the Calibration curves. Four compounds in plasma and brain homogenate samples within the scope of 0.05–5 μg mL^−1^ has excellent linearity (Table 2).

**TABLE 2:** Calibration curves of compounds in plasma and brain.

	**Compounds**	**Internal standard**	**Calibration curves**	**R** ^**2**^
Plasma	CUR	ILE	Y = 2.7723X-0.0059	0.9999
	DHC	ILE	Y = 1.8494X-0.0062	0.9999
	PIP	ILE	Y = 3.3145X-0.0285	0.9998
	CUR	PIP	Y = 1.7085X-0.0067	0.9999
	DHC	PIP	Y = 1.1370X-0.0034	0.9999
	ILE	PIP	Y = 1.1857X-0.0006	0.9998
Brain	CUR	ILE	Y = 3.3383X-0.0625	0.9998
	PIP	ILE	Y = 3.3163X-0.0011	0.9999
	CUR	PIP	Y = 1.9622X-0.0274	0.9998
	ILE	PIP	Y = 1.3054X-0.0017	0.9999

In plasma samples, LOD of CUR, PIP, ILE, DHC were 8.7, 7.4, 9.3, and 12.7 ng mL^−1^, and the LOQ were 29.7, 25.0, 31.7, and 43.2 ng mL^−1^, respectively. In addition, the recoveries of four compounds in this method were between 102.2 and 105%. The intra-day and inter-day precision were less than 3.3 and 1.8%. In the brain homogenate samples, the LOD of CUR, PIP and ILE were 8.8, 7.9, and 10.0 ng mL^−1^, and the LOQ were 29.4, 26.3, 33.3 ng mL^−1^, respectively. The recoveries of the methods ranged from 101.3 to 104.6%, and the intra-day and inter-day precision were all less than 2.5 and 1.4% (Table 3).

**TABLE 3:** Intra-day precision and Inter-day precision of plasma and brain.

	**Compounds**	**Concentrations (μg·mL^−1^)**	**Mean recoveries±sd (%)**	**Intra-day precision (%)**	**Inter-day precision (%)**
Plasma	CUR	0.05	104.2 ± 2.3	1.2	1.3
		0.2	102.6 ± 0.9	1.0	0.3
		1	102.4 ± 0.5	0.4	0.4
	PIP	0.05	105.0 ± 3.0	3.3	0
		0.2	102.4 ± 1.7	1.3	1.4
		1	103.4 ± 1.4	0.8	1.3
	ILE	0.05	103.7 ± 3.2	1.8	1.8
		0.2	102.2 ± 1.8	1.2	1.4
		1	102.7 ± 0.8	0.8	0.2
Brain	CUR	0.05	102.6 ± 2.8	2.5	0.7
		0.2	101.4 ± 1.2	0.6	1.2
		1	101.3 ± 1.6	1.6	0.7
	PIP	0.05	104.3 ± 1.8	1.1	0.6
		0.2	102.7 ± 1.4	0.3	1.4
		1	103.7 ± 1.7	1.4	1.1
	ILE	0.05	104.6 ± 1.7	1.1	0.3
		0.2	103.2 ± 1.2	0.9	0.8
		1	102.7 ± 2.3	2.2	1.4

The recoveries of CUR, PIP, ILE, DHC in plasma and CUR, PIP, ILE in brain homogenate were all over 100% after repeated freezing-thawing for three times, demonstrating that repeated freezing-thawing had no significant effect on the drugs in samples. The recoveries of compounds in plasma and brain homogenate samples were more than 100% after 15 days of cryopreserved at −80°C. The recoveries of CUR and DHC in plasma samples were significantly reduced to less than 30% after being kept at room temperature for 12 h. While, the recoveries of PIP and ILE were lower than 75 and 90% after being kept at room temperature for 12 h. In brain homogenate samples, the recovery rate of CUR decreased significantly (lower than 15%) after 12 h placement at room temperature, while the recovery of PIP and ILE only showed slight decrease.

The peak area of the compounds, which were submitted to sample pretreatment, did not change after being kept at room temperature for 12 h.

#### Qualitative Analysis of CUR Metabolin

Using negative ion scanning, the molecular ion peak of this metabolite in Q1 scanning was m/z 369, which was consistent with that of DHC. The retention time of the metabolite was 3 min, which was the same as that of the DHC standard. The UV absorption spectra of the metabolite scanned by DAD were also consistent with that of the DHC standard. The collected metabolites were scanned by MS anion Q1 showing m/z 369, which was consistent with DHC. Therefore, the metabolite is most likely DHC.

#### Pharmacokinetic

The plasma concentrations of the compounds were shown in Table 4. After combined administration of CUR and PIP, C_max_ of DHC increased by 23%, T_max_ decreased by 15.4%, T_1/2_ increased by 2.4%, AUC_(0-tn)_, Vd and CL decreased by 7.2, 69.2 and 70%, respectively, compared with those of single CUR administration. However, statistically, analysis of variance between DHC concentration after CUR alone administration and combined administration of CUR and PIP showed that *p* value was greater than 0.05, indicating that there was no significant difference between CUR administration alone and combined administration of CUR and PIP.

**TABLE 4:** Blood concentrations of DHC, PIP and ILE.

**Time (min)**	**Mean concentrations ±sd (μg·mL^−1^)**				
	**Group of A**	**Group of B**		**Group of C**	
	**DHC_A_**	**DHC_B_**	**PIP**	**DHC_C_**	**ILE**
20	6.1 ± 3.2	7.5 ± 1.1	1.4 ± 0.4	9.2 ± 2.4^*#^	1.3 ± 0.8
40	3.5 ± 0.9	4.0 ± 0.6	2.1 ± 0.7	6.3 ± 0.7^*#^	1.6 ± 0.1
60	1.8 ± 0.5	2.6 ± 0.5	4.2 ± 0.7	4.8 ± 0.7^*#^	2.0 ± 0.5^**^
90	1.4 ± 0.5	2.0 ± 0.4	2.6 ± 0.5	4.0 ± 1.2^*#^	2.8 ± 0.9
150	1.2 ± 0.7	1.0 ± 0.3	2.4 ± 0.9	3.8 ± 0.7^*#^	5.5 ± 1.9^**^
210	1.1 ± 0.3	0.6 ± 0.2	2.2 ± 0.5	2.6 ± 0.1^#^	3.4 ± 0.5^**^
270	0.9 ± 0.4	0.25 ± 0.03	1.7 ± 0.4	1.4 ± 0.7	2.2 ± 0.4
330	0.8 ± 0.4	0.15 ± 0.03	1.4 ± 0.2	0.6 ± 0.2	1.2 ± 0.8

*The results of variance analysis with C_DHC_ of A showed a significant difference (*p* < 0.05).

#The results of variance analysis with C_DHC_ of B showed a significant difference (*p* < 0.05).

**The results of variance analysis with C_PIP_ of B showed a significant difference (*p* < 0.05).

Group of A: Group of CUR.

Group of B: Group of CUR and PIP.

Group of C: Group of CUR and ILE.

After combined administration of CUR and ILE, C_max_ of DHC increased by 50.8 and 22.7%, T_max_ decreased by 30.8 and 18.2%, T_1/2_ increased by 3.6 times and 3.5 times, and AUC_(0-tn)_ increased by 1.2 times and 1.4 times, respectively, compared with CUR alone and combined administration with PIP. Vd was reduced by 58.6% compared with CUR alone administration and increased by 34% compared with CUR and PIP combined. CL was reduced by 77.4 and 24.6% compared with CUR alone and combined with PIP, respectively (*p* < 0.05). The pharmacokinetic parameters of the DHC were shown in Table 5.

**TABLE 5:** Pharmacokinetic parameters of DHC.

**Pharmacokinetic parameters**	**DHC _A_**	**DHC _B_**	**DHC _C_**
C_max_ (μg·mL^−1^)	6.1 ± 3.2	7.5 ± 1.1	9.2 ± 2.4^*^
T_max_ (min)	26.0 ± 5.5	22.0 ± 4.5	18.0 ± 2.7^*^
T_1/2_ (min)	24.8 ± 9.3	25.4 ± 6.3	114.3 ± 31.2^*^
AUC_(0-tn)_ (μg·min·mL^−1^)	456.6 ± 54.6	423.4 ± 25.8	1,006.0 ± 63.8^*^
Vd (mL)	9,087.6 ± 302.9	2,802.6 ± 133.4^*^	3,751.4 ± 165.6^*^
CL (mL·min^−1^)	233.2 ± 7.8	70.0 ± 3.3^*^	52.7 ± 2.3^*^

*The results of variance analysis with A showed a significant difference (*p* < 0.05).

Group of A: Group of CUR.

Group of B: Group of CUR and PIP.

Group of C: Group of CUR and ILE.

ILE were compared with PIP at the same dose, the C_max_ increased by 32.5%, T_max_ increased by 1.7 times, T_1/2_ decreased by 40.2%, and AUC_(0-tn)_ increased by 32.1% (Table 6).

**TABLE 6:** Pharmacokinetic parameters of PIP and ILE.

**Pharmacokinetic parameters**	**PIP**	**ILE**
C_max_ (μg·mL^−1^)	4.2 ± 0.7	5.5 ± 2.0
T_max_ (min)	68.0 ± 10.9	184.0 ± 23.0
T_1/2(el)_ (min)	167.2 ± 35.4	100.0 ± 17.2
AUC_(0-tn)_ (μg·min·mL^−1^)	701.0 ± 50.8	926.2 ± 94.8

**FIGURE 4:**
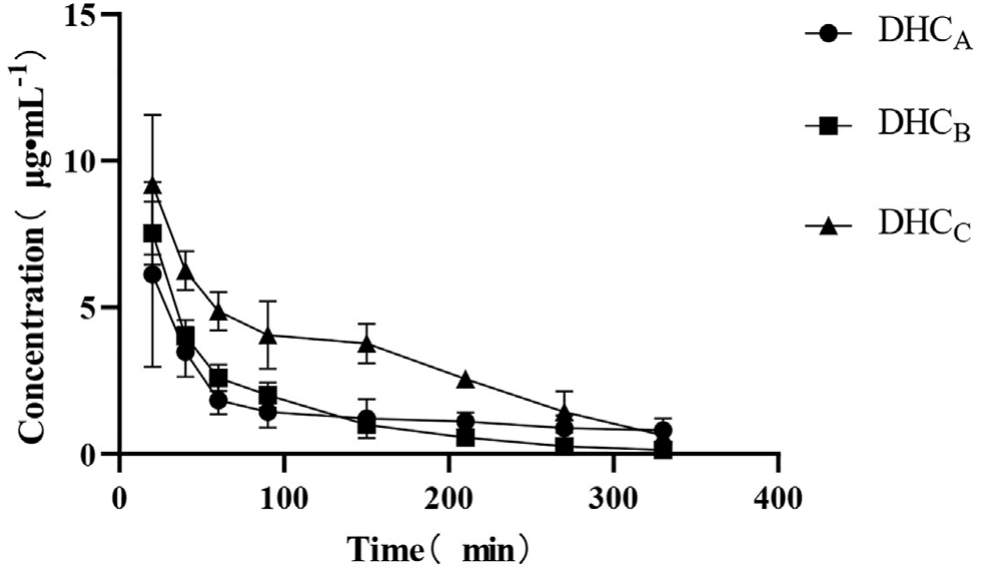
Drug-time curve of DHC. Group of A: Group of CUR, Group of B: Group of CUR and PIP, Group of C: Group of CUR and ILE.

**FIGURE 5:**
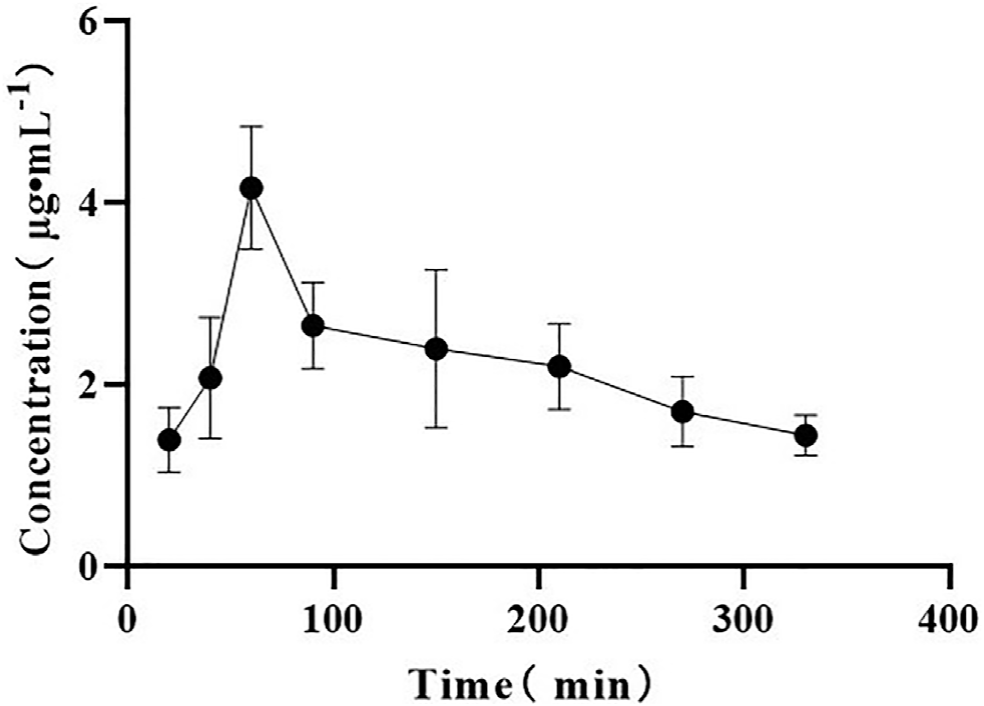
Drug-time curve of PIP.

**FIGURE 6:**
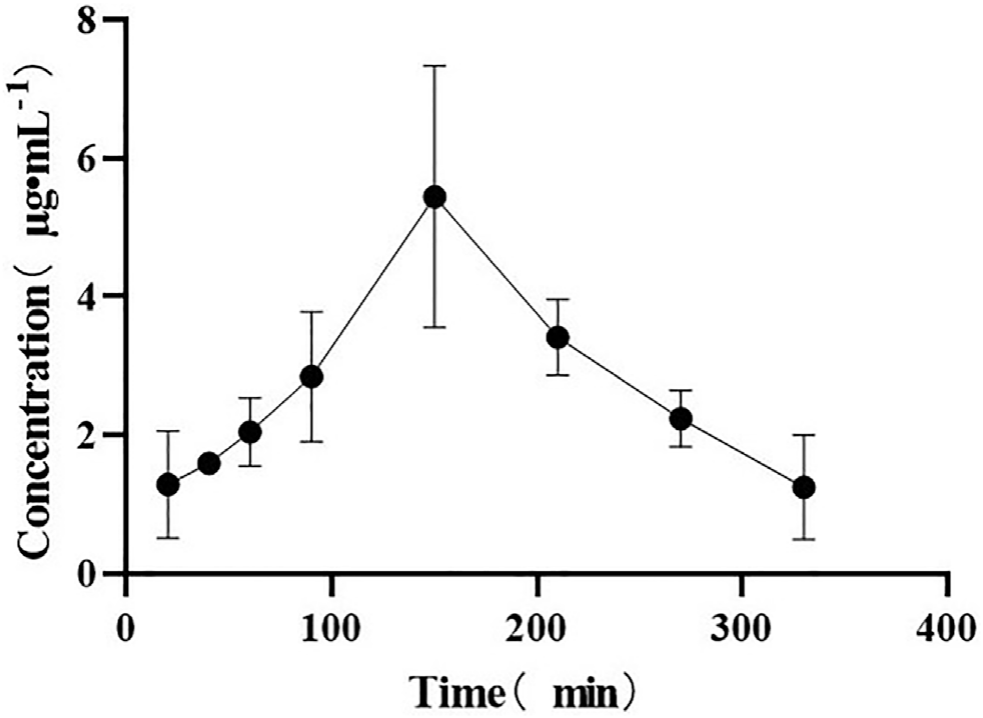
Drug-time curve of ILE.

On the drug-time curve (Figures 4–6), the combined administration of PIP and CUR had no obvious effect on plasma concentration of DHC, while the combined administration of ILE and CUR could increase plasma concentration of DHC (*p* < 0.05). In the experiment of repeated administration, PIP and ILE could both increase the plasma concentration of DHC. PIP and ILE could be detected in brain tissue, whereas, CUR and DHC could not be detected (Table 7).

**TABLE 7:** Drug concentrations of plasma and brain after multiple doses.

**Compounds**		**Mean concentrations±sd (μg·mL^−1^)**	**CV (%)**
Plasma	DHC_I_	1.4 ± 0.6	42.8
	DHC_II_^*^	4.6 ± 1.8	39.1
	PIP	2.4 ± 0.5	20.8
	DHC_III_^*^	8.7 ± 2.5	28.7
	ILE	2.8 ± 0.3	10.7
Brain	CUR	ND	—
	DHC	ND	—
	PIP	2.1 ± 0.1	4.8
	ILE	1.6 ± 0.2	12.5

*The results of variance analysis with A showed a significant difference (*p* < 0.05).

Group of I: Group of CUR.

Group of II: Group of CUR and PIP.

Group of III: Group of CUR and ILE.

#### Absorption and Metabolism of Curcumin in SD Rats

Chromatographic peak of its metabolite DHC could only be detected from plasma samples collected from the rat tail vein after 20 min intragastric administration of CUR, but not CUR. The chromatographic peaks of CUR could be detected in plasma collected at the same time after intravenous injection of CUR, indicating that most of the CUR might be reduced to DHC during digestion and absorption through the gastrointestinal tract.

CUR could be transformed into DHC after incubating CUR with small intestine of SD rats for 2 h, demonstrating that CUR could be reduced to DHC by NADPH mediated by gastrointestinal microorganisms through gastrointestinal digestion and absorption.

## Discussion

By optimizing the chromatographic conditions, a method for simultaneous detection of CUR, DHC, PIP and ILE was established. The four compounds could be completely eluted by isometric elution with mobile phase of 2% HAC(in water): ACN = 42:58.

Because CUR and PIP have antioxidant properties and are easy to decompose under light, strong acid and oxidant should not be used during the pre-treatment process. Additionally, long time process is also unsuitable because of drug loss. In this experiment, a protein precipitant with high recovery rate was developed by using a simpler sample pretreatment method, which could fully precipitate the plasma protein and release the drug. The method is simple and efficient. The peak area of the treated samples remained unchanged after being placed at room temperature for 2 h, indicating that the placement at room temperature had no effect on the experimental results during the period from the treatment of samples to the detection. The protein Precipitation Solution is not only suitable for the treatment of plasma samples, but also has a high recovery rate for the pretreatment of homogenate samples of brain tissue.

This study was to explore the effect of PIP and ILE on plasma CUR concentration. The chemical property of the three drugs is relatively close, therefore PIP and ILE conform to the standards as internal standard substance. Internal standard method is adopted to reduce the effect of the experimental operation error, the method can detect the concentration of the three drugs at the same time, meeting the needs of the experiment.

The effect of protein precipitation solution on spike recovery of CUR in blood plasma was studied. The results showed that mixture of ACN and MeOH increased the recoveries that were several times higher than that of ACN or MeOH alone, and the recoveries increased with the increase of ACN proportion in the mixture of these two solvents. The protein precipitation solution of 90% ACN and 10% MeOH (1% HAc) has high recovery, and is simple and efficient, compared with other methods. The mechanism is not yet clear, but it is suspected to be the synergistic effect between organic solvents. When acetonitrile is used alone to precipitate plasma proteins, the structure of the precipitated proteins is relatively compact and easy to aggregate, resulting in the drug being wrapped by the proteins and therefore reducing the recovery rate. While methanol was used to precipitate plasma protein alone, the structure of the precipitated protein was relatively loose, but the effect of precipitating protein was not as good as that of acetonitrile alone. In addition, acids promote denaturation and hydrolysis of proteins to enhance extraction efficiency. Therefore, the combined use of the three organic solvents can improve the efficiency of protein precipitation.

The results of methodological verification showed that the preprocess of plasma and brain homogenate samples from SD rats had good stability and reproducibility. Due to the transformation of CUR under light, the recovery rate of CUR in the samples decreased significantly after being placed at room temperature for 12 h. After sample treatment and repeated freezing and thawing have little effect on drug recovery, so the repeated thawing of samples during the test period after sample treatment have no effect on experimental results. During the experiment, the samples can be stored at −80°C, and thawed in batches before testing to avoid staying at room temperature for too long.

The established HPLC-UV method for simultaneous detection of CUR, PIP, ILE and DHC in biological samples was stable with the intra-day and inter-day coefficients of variation were less than 3.3%. It has the advantages of simple operation and high recovery, which can meet the requirements of the following experiment.

80% DMSO and 20% EtOH were used as solvent for intragastric administration in this study, and the dosage was 2.5 times less than administering the drug directly, but the bioavailability was 29 times higher. During the experiment, SD rats were administered with DMSO as solvent of intragastric administration for five consecutive days, and no adverse reactions occurred in the experimental animals. Therefore, it is safe to use DMSO as solvent of intragastric administration in animal experiments.

Our results showed that PIP did not significantly affect the plasma concentration of DHC, which might be due to the dose-dependent effect of PIP. When the ratio of CUR and PIP dose was different, the bioavailability enhancer effect of PIP was also different. The combination of ILE and CUR could effectively increase the bioavailability of CUR, and its blood concentration was significantly different from that of CUR alone or CUR combined with PIP. Therefore, ILE as a piperine derivative, also has the pharmacological effect of bioavailability enhancer. Compared with natural drugs, ILE is a synthetic drug with advantages of simple synthesis process and low cost. In addition, it is a drug with independent property rights in China. Accordingly, the discovery of its new uses is conducive to the clinical promotion of CUR and ILE.

In repeated dosing experiments, the initial loading dose helped the blood concentration reach C_max_ more quickly, followed by continuous dosing with half dose. The results showed that PIP and ILE significantly improved the bioavailability of CUR, which can provide a reference for the dosage and appropriate interval time of CUR, PIP and ILE administration.

The Drug-time curve of DHC showed the characteristics of double exponential function. The weight of SD rats in this experiment was 330 ± 30 g, so, the calculated blood volume was 6.4 ml/100 g. The blood volume of SD rats used in this experiment was 21.1 ml, which was far less than the VD value of DHC. Therefore, it can be determined that DHC is distributed in other tissues besides blood. According to the drug time curve of DHC and the analysis of pharmacokinetic parameters, the elimination atrioventricular model of DHC conforms to the characteristics of two-compartment atrioventricular model.

It has been reported in many literatures that PIP can improve the blood concentration of CUR, and the generally accepted mechanism is that PIP, as an inhibitor of glucuronic acid, blocks the metabolism of CUR, thus increasing the blood concentration. In this experiment, after the combined use of PIP and CUR, the T_1/2_ of DHC (a metabolite of CUR), was prolonged and the CL was decreased, which was consistent with this mechanism. As a derivative of PIP, ILE had similar pharmacokinetic parameters of DHC in plasma after the combination use of ILE and CUR with prolonged T_1/2_ and decreased CL. The results demonstrate that ILE may also inhibit the metabolism of CUR and DHC by inhibiting the glucuronidation or glucosidation pathway.

This work also showed that only DHC was detected in the plasma after orally administration of CUR. In addition to the objective factors that may be the insensitivity of the established method, it is speculated that the absorption and transformation enzymes in the liver, small intestinal mucosa or gastrointestinal microorganisms might be involved. Even though the CUR in plasma can be converted into DHC after injection of CUR from the tail vein, the reduction rate was much slower than that of oral administration. Therefore CUR was reduced to DHC when absorbed by the gastrointestinal tract. To study the absorption and metabolism of CUR in small intestine, the small intestine homogenate of SD rats was incubated with CUR at 37°C (pH 7.2) for 2 h after removal of microorganisms. As a result, however, DHC was not detected in this experiment, indicating that enzymes in the small intestinal mucosa have no significant effect on the conversion of CUR to DHC. *In vivo* experiments, the CUR administrated from gastrointestinal tract could be converted to DHC, indicating that gastrointestinal microorganisms might play a critical role in the metabolism of CUR to DHC.

In the previous animal experiments, large doses were needed to achieve the detection of blood CUR. The reasons might be attributed to the low bioavailability of CUR, and the reduction of CUR to form DHC by NADPH mediated by gastrointestinal microorganisms as well.

CUR and DHC were not detected in brain tissue. It might be because DHC could not cross the blood brain barrier (BBB).

## Conclusions

A HPLC-UV method for determination of CUR, PIP, ILE and DHC simultaneously. The method was easy to operate and good reproducibility. ILE could improve the bioavailability of CUR. Both PIP and ILE could significantly improve the bioavailability of CUR after repeated administration. In addition, when most of CUR could be reduced to DHC after orally administration, which might be mediated by gastrointestinal microorganisms.

## Data Availability

The original contributions presented in the study are included in the article/supplementary material, further inquiries can be directed to the corresponding author.
